# Lobular Capillary Hemangioma of the Buccal Mucosa: A Rare Presentation

**DOI:** 10.7759/cureus.65904

**Published:** 2024-07-31

**Authors:** Padmashri P Kalmegh, Alka Hande

**Affiliations:** 1 Oral and Maxillofacial Pathology, Sharad Pawar Dental College and Hospital, Datta Meghe Institute of Higher Education and Research, Wardha, IND

**Keywords:** port wine stain, vascular tumors, venous malformation, lobular capillary hemangioma, capillary malformation

## Abstract

Hemangiomas are benign vascular tumours of the head and neck. Lobular capillary hemangioma (LCH) is a common, acquired proliferative reaction in vascular tissue. It has female predilection, and peak incidence occurs in adolescents and young adults. Histopathologically, it is characterised by nodular proliferation of capillary-sized vessels lined by endothelial cells with plump nuclei. The capillary lumen shows the presence of numerous erythrocytes. To distinguish this lesion from other vascular lesions, a precise diagnosis is required. The majority of oral hemangiomas will regress without any treatment. If these tumours continue into adulthood, it may lead to difficulty in speech and swallowing. This case report presents an atypical manifestation of LCH of the buccal mucosa in a 51-year-old male patient.

## Introduction

Vascular lesions are the most prevalent congenital and neonatal anomalies. Vascular lesions that develop in the oral and maxillofacial regions can lead to both functional and aesthetic concerns. Vascular lesions are derived from blood vessels and lymphatics with different clinical and histological features and treatments [1]. Hemangioma is benign neoplastic vascular proliferation that results from a derangement in angiogenesis with the exuberant proliferation of vascular elements due to an imbalance between angiogenic and angiostatic forces [2]. It may be present at the time of birth or in later life. A prevalence analysis indicates that oral hemangiomas make up 14.3% of all benign vascular lesions [3]. They are more prevalent in white races than in black. Very few cases develop intravenously, with the majority occurring in the soft tissues (muscle, mucosa, and skin) [4]. They have two subtypes of hemangioma: capillary hemangioma (CH) and cavernous hemangioma. A capillary type appears as a flat lesion in contrast to a cavernous hemangioma, which appears as an elevated lesion and consists of large dilated vascular channels filled with blood [5]. The given case has an atypical presentation in that the lesion is observed on the buccal mucosa rather than the gingiva, and the patient is a 51-year-old male, compared to the female prevalence seen in the second to third decade.

## Case presentation

**Figure 1:**
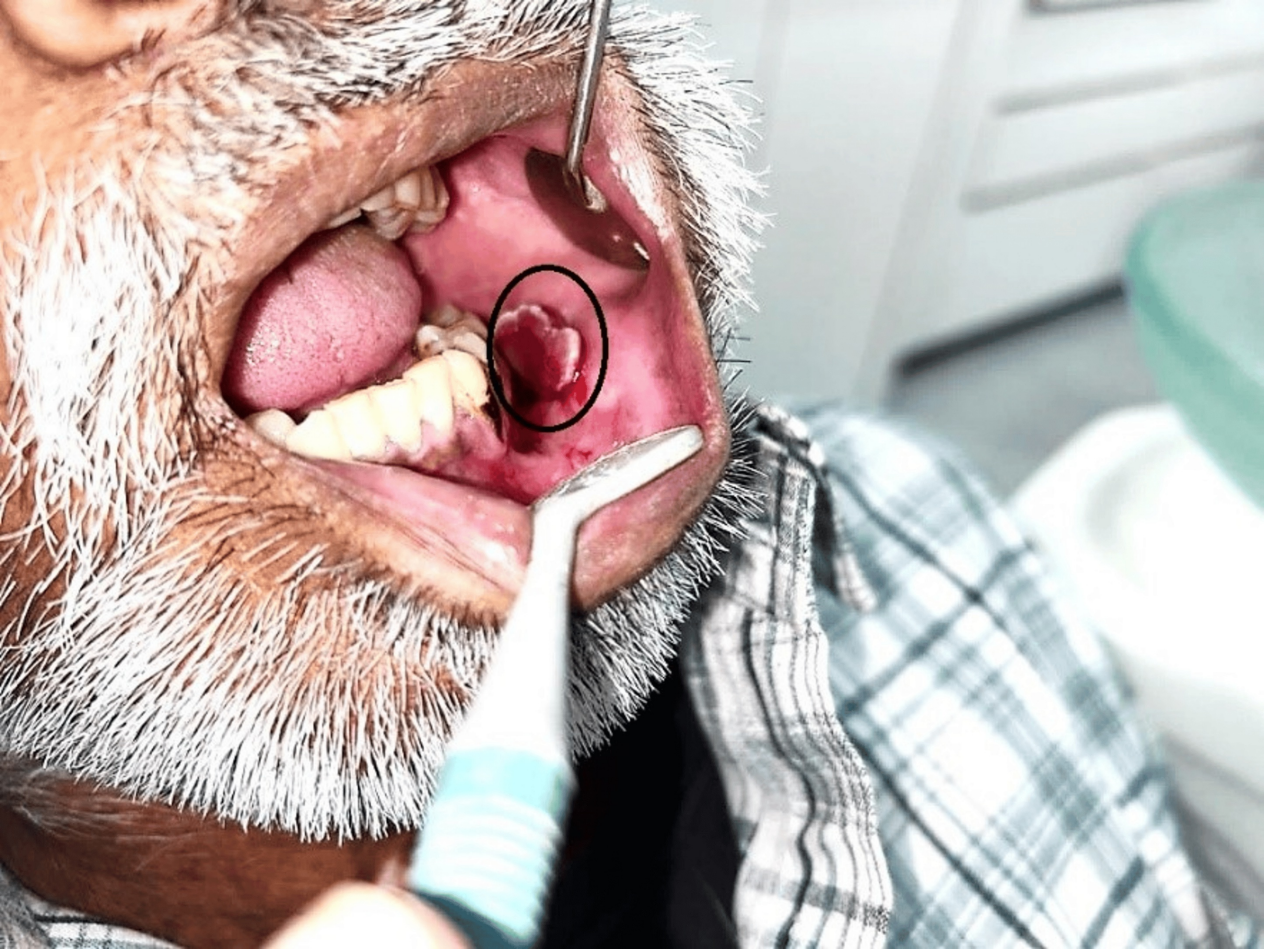
Intraoral lesion on the buccal mucosa

A 51-year-old male reported swelling over the left buccal mucosa for two to three months. A patient was alright three months back. He noticed swelling on the buccal mucosa. Initially, the swelling was 0.6 x 0.3 cm in dimension and increased to a present size of 2.2 x 1.5 cm. No extraoral extension of the swelling was noted. On intraoral examination, the swelling was solitary, sessile, reddish pink with distinct borders and a smooth surface, which extended from mesial of 35 to distal of 36 (Figure 1).

Palpation revealed a painful, soft swelling that blanched when pressure was applied. The examination of the other soft and hard tissues was normal. No lymph nodes were palpable. The patient had poor oral hygiene. There is no history of trauma as reported by the patient. In the fifth decade of life, a very rare occurrence of CH on the buccal mucosa is described in this case report. Although the lesion in this particular case was clinically diagnosed as papillomatous, the lesion was determined histopathologically to be lobular CH (LCH).

**Figure 2:**
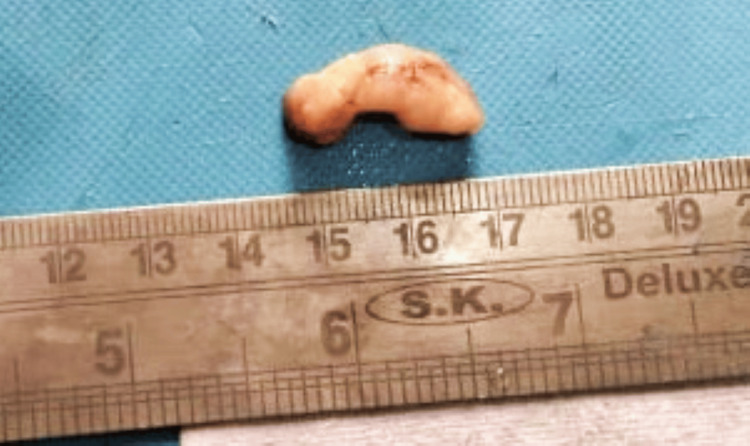
Macroscopic specimen

Macroscopically, we received single, oval, whitish, soft-firm bits of specimen measuring 1.9 x 1.6 x 0.8 cm (Figure 2).

**Figure 3:**
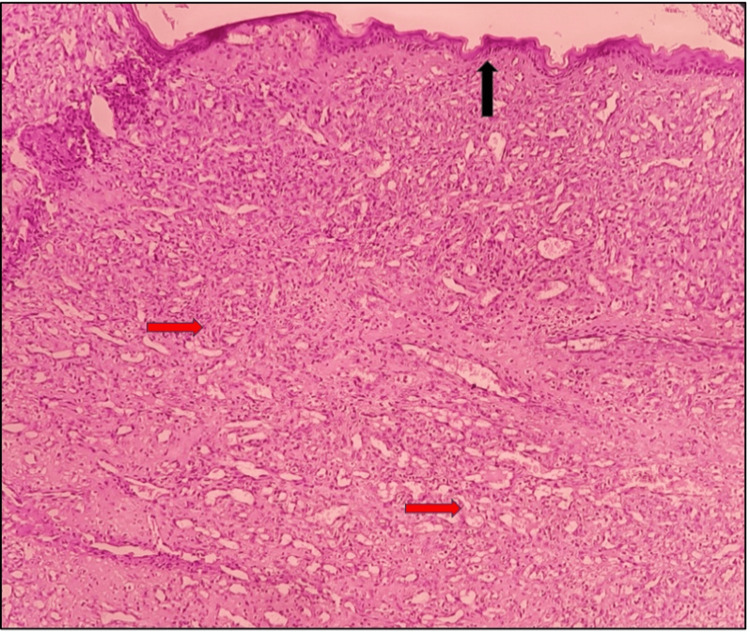
Microscopic presentation: hematoxylin and eosin-stained section under low-power view (10×)

On microscopic examination, the low-power view shows the hematoxylin and eosin (H&E) stained tissue section comprising of overlying stratified squamous epithelium (black arrow) and underlying connective tissue stroma (red arrow) (Figure 3).

**Figure 4:**
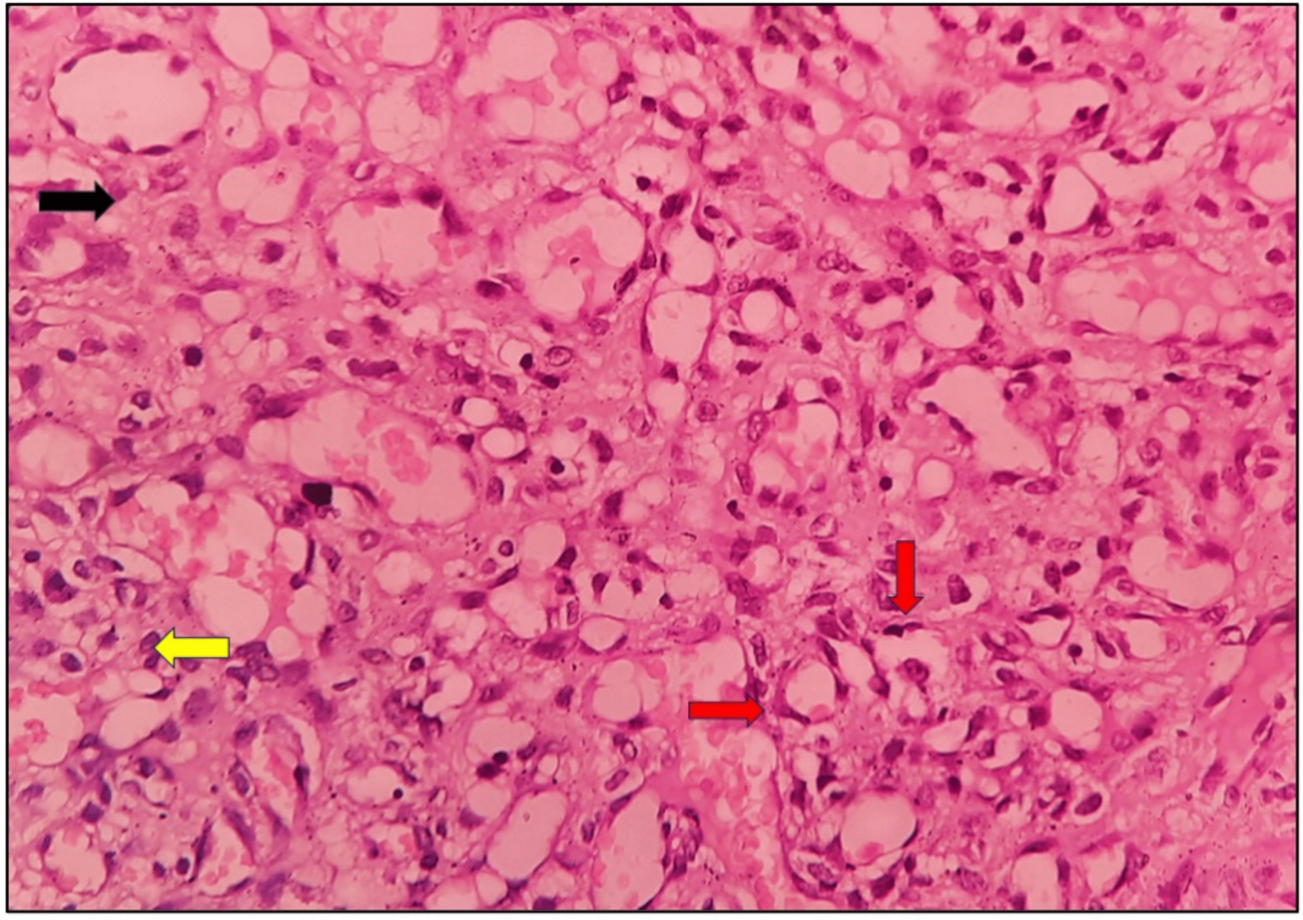
Microscopic presentation: hematoxylin and eosin-stained section under high-power view (40×)

On microscopic examination, a high-power view shows the presence of a connective tissue stroma (black arrow). There are numerous engorged endothelial cell-lined capillaries are present. In many places, capillaries are in a lobular pattern (red arrow). There is a presence of inflammatory cell infiltration (yellow arrow). Histopathological features are suggestive of LCH (Figure 4).

In this case, surgical excision of the lesion was done followed by the six-month postoperative follow-up.

## Discussion

Hemangiomas are benign lesions that can arise congenitally or develop during the first few weeks of life. LCH are rare lesions of the oral cavity [3]. Haemangiomas are hamartomas lesions [6]. It does not transform into malignancy [5]. The first case of the hemangioma was reported by Liston in 1843 [4]. To describe the underlying histology counterpart, LCH was first used in 1980. A contributing factor to the uncertainty in the literature is the major change in the definition of this term throughout time, from granulation tissue, reactive hyperplastic mucosal lesion, to a benign vascular tumour. Hemangioma presents in the LCH and the non-LCH forms, having unique clinical features and histopathological and pathophysiological characteristics [7]. Although cases of congenital hemangioma are frequently present at birth, it may become more obvious later in life [5].

Clinically, it is a painless solitary nodule or sessile papule with an ulcerated, bleeding surface that is smooth or lobulated. The soft tissue mass is compressible and non-pulsating. These lesions may vary in dimension from millimetres to centimetres ranging [8]. It more commonly affects females than males (3:1) [3]. Higher incidences are seen during pregnancy, which could be linked to an increase in progesterone and oestrogen levels [9]. It is most commonly seen in the second and third decades of life [10]. It may manifest in the fifth decade of life as seen in the present case. Angiogenic growth factors such as “vascular endothelial growth factor” and “β-fibroblast growth factor” are more prevalent under these types of conditions [8]. The lesion can be pink, red, or purple in colour. When pressure is applied, the lesion blanches, and bleeding can happen on its own or in response to moderate trauma [5]. Studying 31 cases of intraoral CH, according to Matsumoto et al., the buccal mucosa had the highest number of lesions, followed by the tongue, lip, gingiva, and palate [11]. They may be cutaneous, intramuscular, and intra-osseous [5]. Trauma in the past may be the cause of extra gingival LCH, while chronic oral irritants such as overhanging restorations, poor oral hygiene, and even hormonal changes may cause gingival LCH [12].

Based on histopathological features, hemangiomas are categorised as capillary, cavernous, or mixed. CH is a collection of tiny, localised lesion, capillary-sized vessels with thin walls. A discontinuous layer of pericytes and reticular fibres surrounds the vessel, which is lined by a single layer of plump endothelial cells [3]. “Port-wine stain” is the most typical presentation. Because of the typical arrangement of blood capillaries in a lobular form, it is often referred to as "LCH" [8]. The average vessel diameter of the lobular area is approximately 9 microns [7]. Cavernous hemangiomas are deep, asymmetrical, blood-filled dermal pathways made up of cavernous veins or sinusoids with thin walls that are divided by a scant layer of connective tissue [3].

Both the terms capillary and cavernous hemangioma are no longer used, and the lesions are described as superficial, deep, or complex hemangioma depending on the extent of the lesion. Superficial haemangiomas, often known as strawberry hemangiomas, are bright red macular or papular masses that originate from the papillary dermis. Deep haemangiomas, formerly known as cavernous haemangiomas, are masses that appear bluish or comparatively colourless and are found within the reticular dermis or subcutaneous tissue. In the early stages, LCH are very vascular and bleed easily; however, as the lesion matures and turns pink and more collagenous, this bleeding diminishes. At times, fibrous progressing mature lesions can resemble fibromas or develop into them [12]. In addition to squamous cell carcinoma, chronic inflammatory gingival hyperplasia (epulis), “peripheral giant cell granuloma”, “pyogenic granuloma”, and “peripheral ossifying fibroma” are included in the differential diagnosis of hemangiomas [13]. It is relatively infrequent in the oral cavity and is rarely seen by medical professionals [5]. The average diagnosing process takes three months. The likelihood of cutaneous cancer rises if the lesion persists for more than six months [12].

Vascular lesions present difficulty in terms of bleeding and healing. Ulceration after surgery is a typical problem. The current therapy options include endovascular embolisation alone or in conjunction with surgery, intralesional sclerosing agent injection, one to three lasers, and systemic steroids [4]. Recurrence rates vary from 3.7% to 43.5% [12]. A longer follow-up is necessary due to the higher risk of postoperative recurrence associated with CH [3].

## Conclusions

Lobular capillary hemangioma is an uncommon form of angiomatous growth. Oral capillary hemangiomas are small, thin-walled capillary-like vessels with an endothelial cell lining that are infrequently observed in adults and are primarily seen on buccal mucosa. Differential diagnoses might result from variations in clinical presentation and size. As a result, a biopsy is required for a final diagnosis. A lobular proliferation of capillary blood vessels has been identified as the histological characteristic of LCH. The majority of true hemangiomas require no treatment. Excision is the most common treatment, and the prognosis is excellent. Recurrence has been reported. 

The current case report enlightens an exceptionally unusual atypical appearance of lobular capillary haemangioma that enlightens the diagnostic approach, varied histopathological presentation, and treatment modalities.
